# A Scoping Review of School‐Based Programs for Promoting Recently Arrived Immigrant Youth's Positive Adjustment and Well‐Being

**DOI:** 10.1002/jcop.70088

**Published:** 2026-02-10

**Authors:** Metin Özdemir, Brit Oppedal, Sandra Altebo Nyathi, Layan Amouri, Hasnaa Amouri, Sevgi Bayram Özdemir

**Affiliations:** ^1^ Center for Lifespan Developmental Research Örebro University Örebro Sweden; ^2^ Norwegian Institute of Public Health Oslo Norway

**Keywords:** adolescents, effectiveness, immigrants, program evaluation, refugees, school‐based services, social adjustment

## Abstract

This scoping review examines school‐based programs aimed at promoting the adjustment and well‐being of recently arrived immigrant adolescents. Following PRISMA‐ScR guidelines, five databases (Medline, PsycINFO, CINAHL, SCOPUS, ERIC) were systematically searched for studies published since 2000, focusing on interventions implemented in formal school settings for recently arrived adolescents. The review identified 15 studies evaluating outcomes of 17 programs. Most school‐based programs for recently arrived adolescents aimed to promote social‐emotional well‐being, mitigate mental health problems, strengthen resilience and social support, or address trauma‐related symptoms. Around 50% of the reviewed programs had some effects on the intended outcomes. Despite some promising findings, the current literature has several limitations that limit the ability to draw robust conclusions. Future research needs to focus on understanding why, how, and for whom programs lead (or do not lead) to intended outcomes, and on developing effective programs that can be implemented using available resources at school.

The global number of international migrants has increased by 83% over the past three decades, rising from 150 million to 281 million, driven by factors such as climate change, natural disasters, political instability, conflict, and pursuit of economic opportunities (McAuliffe and Oucho 2024). Notably, approximately 13% of these international immigrants are under the age of 18 (UNHCR 2023). Regardless of the reasons for migration, moving to a new country with a different socio‐cultural environment creates additional strains on children and adolescents (McNeely et al. 2017). As schools are the key gathering grounds for all school‐aged minors, school‐based programs have been highly praised to support the adjustment of immigrant and refugee children and adolescents to promote psychosocial adjustment and overcome potential challenges with navigating their new sociocultural environment (Bennouna et al. 2019; McNeely et al. 2017). When mapping school‐based interventions, existing reviews seem to primarily focus on treatment programs that often require specialized training and expertise (e.g., Bennouna et al. 2019; Hettich et al. 2020; Tyrer and Fazel 2014). However, this narrow definition of the target group based on mental‐health needs risks overlooking programs aimed at promoting the adjustment and well‐being of immigrant youth and children. Thus, this scoping review focuses on developing an overview of the school‐based interventions that have been implemented for recently arrived adolescents to promote their positive adjustment and well‐being (excluding physical health promotion programs).

## 1 Migration Experiences during Adolescence

Adolescence is a critical period marked by significant cognitive, emotional, behavioral, and relational changes, in addition to evolving expectations set forth by parents, peers, schools, and even society (McCormick et al. 2011). Although the developmental changes during adolescence, such as building stronger relationships with peers, establishing one's own identity with personal values, moral system, and displaying acceptable social behavior, are universal across ethnic and cultural backgrounds (Jugert and Titzmann 2020; McCormick et al. 2011; Motti‐Stefanidi et al. 2012). Thus, navigating these changes in a new cultural context presents unique and compounded challenges for adolescent immigrants compared to those who migrate in early childhood. These tasks are also influenced and shaped by the cultural and social expectations tied to the larger societal context in which adolescents live (Jugert and Titzmann 2020; McCormick et al. 2011; Motti‐Stefanidi et al. 2012). Therefore, accomplishing these developmental tasks involves more complex adaptation processes among adolescent immigrants because of their limited familiarity with the cultural norms and expectations in the resettlement country, compounded by exposure to a different set of expectations in their home environment (Jugert and Titzmann 2020; Motti‐Stefanidi et al. 2012). Hence, recently arrived immigrant and refugee youth may have more challenges than the native youth in handling developmental tasks, which may hamper their psychological well‐being and ability to adjust. Despite these challenges, studies on immigrant youth in non‐clinical settings have shown that a substantial number of immigrant youth adjust remarkably well in psychological, social, behavioral, and academic domains (Fuligni 1998; Motti‐Stefanidi et al. 2021). However, school‐based programs, may provide especially the recently arrived international immigrants extra support to ease their adjustment process and give them extra tools to handle the challenges of the resettlement process.

Despite the complexity of immigrant youth's experiences and needs, existing research disproportionately emphasizes mental health issues and groups that are at risk for experiencing trauma, such as refugees. Especially the studies on immigrant youth with refugee background underscore the elevated levels of post‐traumatic stress (e.g., Ellis et al. 2008; Heptinstall et al. 2004), depression, and anxiety symptoms (e.g., Derluyn et al. 2008; Karadag and Ogutlu 2020) among the newcomers. Consequently, these findings have driven the development of targeted mental health interventions in clinical, community, and school settings (e.g., Bennouna et al. 2019; Cowling and Anderson 2023; Hettich et al. 2020; Trimboli et al. 2021; Tyrer and Fazel 2014), focusing primarily on treating or reducing symptoms of PTSD, anxiety, and depression. Although it is important to focus on mental‐health needs, particularly among refugee adolescents, this approach risks narrowly defining the challenges faced by immigrant youth as primarily mental health‐related, overlooking the diverse backgrounds of international migrants and their developmental needs. Refugee youth constitute a significant part of the school‐age immigrant population, with approximately 40% of international immigrant minors under 18 years of age classified as refugees (UNHCR 2023). Although they may not be legally designated as refugees, many other recently arrived youth have faced experiences of displacement, violence, or persecution. Beyond these adversities, both refugee and non‐refugee immigrant youth encounter significant developmental challenges, including navigating new cultural contexts, acquiring a new language, forming peer relationships, and achieving academically. Successfully navigating these challenges may not only improve their positive psychological development and functioning but also mitigate the risks of developing internalizing and externalizing problems. Moreover, a narrow emphasis on trauma and pathology overshadows the strengths and resilience that many immigrant youth display despite their limited resources and disadvantaged circumstances (Motti‐Stefanidi et al. 2021; Özdemir and Bayram Özdemir 2020). In fact, a systematic review highlights that the psychosocial needs of refugee children and adolescents extend beyond mental health, encompassing social support, security, cultural understanding, and education (Nakeyar et al. 2018). Yet these broader developmental and acculturative needs remain sparsely explored, leaving many migrant youth underserved by current interventions. Addressing these needs requires a more holistic approach, recognizing the heterogeneity of immigrant experiences and providing support that may promote their adjustment and well‐being in addition to addressing their mental‐health needs.

## 2 Overview of Existing Reviews

Several review and meta‐analysis studies attempted to compile evidence on the effectiveness of interventions targeting recently arrived adolescents and children (e.g., Bennouna et al. 2019; Cowling and Anderson 2023; Giles et al. 2024; Hettich et al. 2020; Martinez et al. 2024; Sreekala et al. 2025; Thabet et al. 2023; Trimboli et al. 2021; Tyrer and Fazel 2014). However, most of these published reviews used a narrow definition of the immigrant populations by exclusively focusing on refugees (UNHCR 2023). Although a few reviews adopted a relatively broader definition encompassing forced migrants or including asylum seekers in the resettlement country (e.g., Bennouna et al. 2019; Thabet et al. 2023), the target group definitions of these studies are still not inclusive. In addition, a common theme of the existing reviews is their exclusive emphasis on treatment and specialized interventions focusing on mental health difficulties (Cowling and Anderson 2023; Hettich et al. 2020; Trimboli et al. 2021; Tyrer and Fazel 2014), rather than universal and promotive interventions. Reviews that focused on school‐based interventions either included only studies conducted in high‐income countries (Bennouna et al. 2019) or studies on refugees within their first year of arrival (Bennouna et al. 2019). Some reviews included school‐based interventions alongside other contexts such as clinics, home, community, and refugee centers (Cowling and Anderson 2023; Giles et al. 2024; Trimboli et al. 2021; Tyrer and Fazel 2014). A recent review by Martinez et al. (2024) focused exclusively on school‐based interventions; however, its primary focus was to map the characteristics of available mental health programs using the typology developed by the National Center for School Mental Health, which categorizes interventions into Tier 1–3 and multi‐tiered systems of support. There were also reviews of evidence about the interventions exclusively focusing on unaccompanied minors (Demazure et al. 2018). Overall, these reviews generally conclude that the quality of existing studies is low, with most findings based on pilot studies and research with low sample sizes. Despite these limitations, the reviews also conclude that various approaches such as cognitive behavioral therapy (CBT), trauma‐focused CBT, narrative exposure therapy, and eye‐movement desensitization and reprocessing therapy have shown some promising effects on reducing post‐traumatic stress symptoms, psychological distress, and depressive symptoms (e.g., Bennouna et al. 2019; Cowling and Anderson 2023; Hettich et al. 2020; Trimboli et al. 2021). In summary, none of these reviews exclusively focused on school‐based interventions to promote social and academic adjustment and psychological well‐being among immigrant adolescents undergoing the resettlement process.

## 3 The Current Study

Although several studies reviewed existing evidence about preventive interventions for immigrant children and adolescents, most of these reviews conceptualized the needs of recently arrived adolescents as being predominantly mental‐health related, overlooking the complex interplay between acculturative and developmental challenges (Jugert and Titzmann 2020; Motti‐Stefanidi et al. 2012). This limited perspective has created a significant gap in understanding how schools can effectively support these students' multifaceted needs in other domains through programs that can be implemented by existing school staff. The current study employs a scoping review methodology because it allows for a broader examination of programs with diverse outcomes related to recently arrived immigrant adolescents' adjustment and well‐being, unlike systematic reviews and meta‐analyses, which typically require more narrowly defined outcome measures and intervention types (Arksey and O'Malley 2005; Munn et al. 2022). Thus, this review examines published studies on school‐based programs targeting recently arrived adolescents (defined as those born in another country who have been in the resettlement country for up to 5 years). It is important to clarify that there is no universally accepted definition of “recently arrived” in the literature. The same group is referred to as “newly arrived” and “newcomer” in different studies or policy documents. Immigrant adolescents typically spend several years navigating key developmental tasks, including identity formation, autonomy, and social integration, within unfamiliar cultural, educational, and familial contexts (Jugert and Titzmann 2020; Titzmann and Silbereisen 2009). Some studies propose specific timelines to identify a person as recently arrived, such as 6 to 7 years to reflect the duration of psychosocial adjustment to the new context (Birman and Trickett 2001). Given that the current review focuses on programs targeting youth aged 12 to 18 and those implemented within formal schooling, we limited the inclusion criteria to adolescents who have lived in the host country for up to 5 years, thereby excluding youth who migrated before reaching school age.

The goals of the scoping review are (1) to provide an overview of the state of the literature on school‐based intervention programs that aim to promote the adjustment and well‐being of recently arrived youth, (2) to review the available evidence for their effectiveness, and (3) to discuss the methodological rigor of the studies and future directions for advancing the literature. While acknowledging the value of clinical and community‐based programs, this review specifically focuses on school‐based interventions because schools can reach students who may face barriers to accessing formal service providers or community‐based programs (McNeely et al. 2017). The current review excludes physical health promotion programs, such as those focusing on weight management, nutrition, hygiene, dental care, or disease prevention.

## 4 Methods

A scoping review method was used to map the extent and range of evidence regarding the effectiveness of the school‐based programs that aim to promote the adjustment and well‐being of the recently arrived immigrant youth (Arksey and O'Malley 2005; Munn et al. 2022). The review process started with the development of the research question, identification of the most relevant databases, systematic search of the databases, screening of studies, selecting papers, extracting data, and summarizing the results (Arksey and O'Malley 2005).

### 4.1 Search Strategy

**Figure 1:**
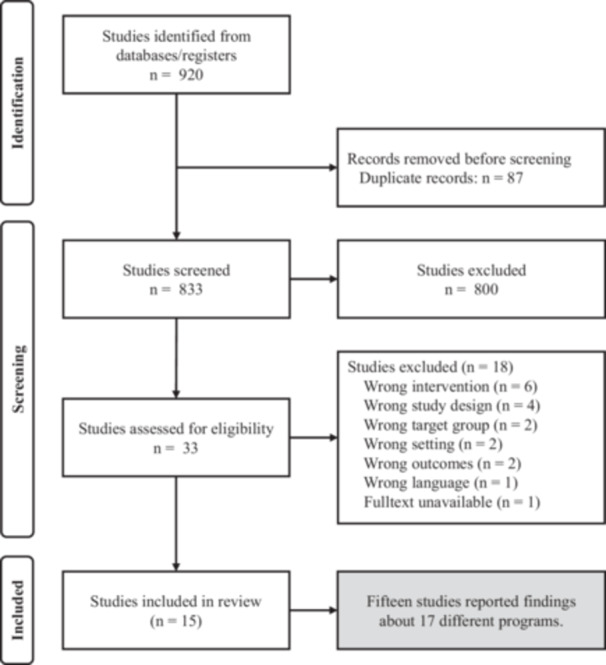
PRISMA flow diagram of the literature search, study screening, and identification.

Five online databases (Medline, PsycINFO, CINAHL, SCOPUS, ERIC) were searched following the Preferred Reporting Items for Systematic Review and Meta‐Analysis Protocols (PRISMA‐P) guidelines (Tricco et al. 2018). The search has been updated to ensure coverage of the most up‐to‐date literature (last update March 3, 2025). A search string was developed to identify intervention studies implemented in the school context for adolescents (age 12 and above) with immigrant or refugee background (including asylum seekers, unaccompanied minors, and displaced persons), excluding physical health promotion programs (see Appendix S1). The search was limited to publications that used quantitative methods, published in peer‐reviewed journals from the Year 2000, and samples in ages between 12 and 22 years of age to cover the groups that were in middle to high school. The search yielded 833 potentially relevant references after removal of the duplicates (see Figure 1). Titles and abstracts of the studies were screened by independent reviewers, and 33 studies were selected for full‐text review. A minimum of two reviewers screened the full texts and identified 15 studies that met the inclusion criteria.

### 4.2 Study Selection

The reference lists extracted from different databases were combined, and duplicates were removed in EndNote software. Next, the list was transferred to the systematic review platform Rayyan (https://www.rayyan.ai) for title and abstract screening. Full‐text review and data extraction were performed in Covidence Systematic Review Software (https://www.covidence.org/). Three authors independently reviewed 10% of the full texts using the inclusion and exclusion criteria and compared the decisions. The agreement rate was very high (*κ* = 0.95). Disagreements were resolved through discussion. A minimum of two authors reviewed the remaining studies to identify the final set of included studies. The studies were retained if they (1) reported findings from a program implementation; (2) focused on programs that were delivered in a formal educational setting, school; (3) reported a program that targeted (or included) adolescents; (4) evaluated a program that included immigrant or refugee participants who recently arrived to the resettlement country; (5) focused on adjustment (e.g., psychological, social, behavioral, academic) and well‐being of youth; (6) were published in a peer reviewed journal. The studies were excluded if the study (1) primarily described program content and/or implementation process; (2) did not include any measure to assess the program outcomes; (3) focused on children or adults; (4) is a review, meta‐analysis, or theoretical/conceptual discussion; or (5) reported outcomes of physical health promotion programs such as weight management, nutrition, hygiene, dental care, or disease prevention (see Table 1 for inclusion and exclusion criteria).

**Table 1:** Inclusion and exclusion criteria used for screening studies.

Inclusion criteria	Reports on the program outcomes Program is delivered in school, or in connection with school Study focuses on adolescents aged 12 and above Participants are recently arrived immigrants or refugees (during the past 5 years) Programs focused on adjustment (e.g., social, psychological, behavioral, and academic) and the well‐being of youth Published in a peer‐reviewed journal
Exclusion criteria	Described a program or implementation process without any outcome, case studies on a single individual, or study protocols Did not include any measures to assess the program outcome Study is on children or adults Review, meta‐analysis, theoretical/conceptual papers Reported outcomes of a physical health‐focused program (e.g., weight management, nutrition, hygiene, dental care, or disease prevention)

### 4.3 Data Extraction and Analysis

Data extraction was performed by three authors working collaboratively on 20% of the selected studies. Once consensus was reached, the remaining studies were reviewed for data extraction. The data extraction process was completed through discussions of specific pieces of information that the reviewers were unsure about. These items amounted to less than 10% of the overall data extracted from the studies. The key information extracted included study characteristics (i.e., authors, title, publication date, and outlet, country/setting of the study), sample characteristics (i.e., sample size, gender distribution, age range, country of origin), evaluation design and measurements, program characteristics (i.e., program type, theory, duration, aims), and findings. In the final stage, the data were analyzed by grouping programs based on key qualities, including evaluation design, program characteristics, and program outcomes.

## 5 Results

### 5.1 Study Selection

The search of the databases yielded 920 potentially relevant references that met the search criteria. Removing 87 duplicates left 833 studies that were screened based on inclusion and exclusion criteria (see Table 1). The review of full‐text articles resulted in 15 studies reporting 17 interventions conducted in 12 countries, most of which were from Europe, North America, and Australia. There was only one study from Lebanon, India, and Turkey, suggesting that most evidence about the school‐based programs comes from high‐income countries. The study characteristics, intervention characteristics, and observed outcomes were summarized in Tables 2 and 3.

**Table 2:** Summary of the geographical location, targeted groups, study design, and overall program effects of the included studies.

References	Country	Origin of participants	*N* (% female)	Age (age range)	Evaluation design	Number of assessments	Overall effect*
Baker and Jones (2006)	Australia	Sudan, Iran, Liberia, Rwanda, Ethiopia, Congo	31 (65%)	13.93 (NR)	Single‐cohort study Pilot/feasibility test	5, during the program duration	No
Cardeli et al. (2020)	United States	Bhutan	34 (46%)	12.94 (12–15)	Single‐cohort study Pilot/feasibility test	2, pre‐ and post‐test	No
Fox et al. (2005)	United States	Vietnam, Cambodia	58 (56%)	10 (6–15)	Single‐cohort study Efficacy study	4 assessments, 1 month before intervention, 4th and 8th week of the intervention, and 1 month after the intervention	Yes
Gormez et al. (2017)	Turkey	Syria	32 (63%)	12.41 (11–15)	Single‐cohort study Efficacy study	2, pre‐ and post‐test	Yes
Hannover et al. (2020)	Germany	Syria, Bosnia, Poland, Lebanon, Afghanistan, Italy	318 (48%)	10.6 (NR)	Non‐randomized controlled Efficacy study	2, pre‐ and post‐test	Yes
Kevers et al. (2022)	Belgium	Syria, Somalia, Afghanistan, Iraq	120 (47%)	9.58 (8–12)	RCT Efficacy study	2 pre‐ and post‐test	Yes
Mom et al. (2019)	Australia	Iraq, Afghanistan, and Pakistan, Africa (Sudan and Somalia), and South East Asia (Burma and East Timor)	32 (52%)	15 (12–18)	Single‐cohort study Efficacy study	2, pre‐ and post‐test	No
Peltonen et al. (2022)							
INSETT program	Finland	Not reported	995 (50%)	14.5 (NR)	RCT	3, pre‐test, 6 and 9 months follow‐ups	No
					Effectiveness study		
PIER program	Finland	Not reported	108 (50%)	14.3(NR)	RCT	3, pre‐test, 6 and 9 months follow‐ups	No
					Effectiveness study		
Quinlan et al. (2016)	Australia	Middle East, East Asia, and Africa	42 (36%)	15.5 (NR)	Non‐randomized controlled Efficacy study	2, pre‐ and post‐test	No
Rousseau et al. (2012)	Canada	Africa, Latin America and the Caribbean, Asia, and other	55 (54%)	14.85 (12–18)	Non‐randomized controlled Pilot/feasibility test	2, pre‐ and post‐test	No
Rousseau et al. (2007)	Canada	Asia, Eastern Europe, South America, the Middle East, and Africa	123 (43%)	NR (12–18)	RCT Pilot/feasibility test	2, pre‐ and post‐test	No
Rousseau et al. (2005)	Canada	Africa, Asia, Europe, South America (Mexico, Central and South America), Canada	138 (41%)	NR (7–13)	Non‐randomized controlled Effectiveness study	2, pre‐ and post‐test	Yes
Spaas et al. (2023)							
CD program	Belgium, Denmark, UK	Mainly born in Syria, Italy, Somalia, and Spain	307(27%)	14.27 (10–19)	RCT	2, pre‐ and post‐test	Yes
					Effectiveness study		
WTS program	Belgium, Denmark, Norway	Mainly born in Syria, Eritrea, Afghanistan, Pakistan, and Romania	251 (47%)	15.07 (11–23)	RCT Effectiveness study	2, pre‐ and post‐test	No
Tubbs Dolan et al. (2022)	Lebanon	Syria	4598 (49%)	NR (5–15)	RCT Effectiveness study	2, pre‐ and post‐test	Yes
Yankey and Biswas (2019)	India	Tibet, India	300 (NR)	NR (13–17)	RCT Effectiveness study	2, pre‐ and post‐test	Yes

* “Yes” refers to when a program had a significant effect in the expected direction on at least one of the measures.

**Table 3:** Summary of the intervention characteristics and the program outcomes.

References	Intervention type	Intervention duration	Intervention language	Facilitator	Intervention outcomes
Baker and Jones (2006)	Prevention‐oriented music therapy	5 weeks	Offered in the majority language	External music therapists	The authors claimed a reduction in externalizing problems, but the mean externalizing problems were actually higher in the last assessment compared to the first. There were no effects on internalizing problems, behavior symptom index, school problems, or adaptive skills.
Cardeli et al. (2020)	Prevention‐oriented trauma systems therapy skill‐based	12 weeks	Only offered intervention in the majority language	External professionals	There was no significant change in depressive symptoms, sense of school belonging, or overall PTSD scores, but a small decrease was observed in avoidance symptoms (*d* = 0.36).
Fox et al. (2005)	Prevention oriented A form of cognitive behavioral interaction Manual based	8 weeks	Offered in the majority language	Teacher	Children's depression scores significantly decreased between screening times 1 and 2, 1 and 3, and 1 and 4.
Gormez et al. (2017)	Prevention‐oriented, protocol‐based, group CBT program Manual based	8 weeks	Offered in the native language	Teacher	Statistically significant reductions post‐intervention in the SCAS total score, intrusive thoughts, and arousal subscales of the PTSD measure, the SDQ emotional problems score, and the number of youth meeting the diagnostic threshold for anxiety and PTSD. However, there was no change in CPTS‐RI avoidance scores, SDQ total score, or subdimensions of conduct problems, hyperactivity, peer problems, and prosocial behaviors.
Hannover et al. (2020)	Promotion‐oriented based on Intergroup Contact theory Manual based	10 weeks	Offered in the majority language	External, university students	The intervention group reported higher self‐efficacy, self‐worth, academic self‐concept, and greater perceived support from peers, both emotionally and academically. However, there was no effect on peer self‐concept or peer networks.
Kevers et al. (2022)	Prevention‐oriented classroom‐based creative arts program	8 weeks	Not mentioned	Teacher	The intervention group rated classroom climate more positively than the control group, though with a small effect size; youth with higher baseline peer relationships saw further improvements; no main effect on mental health outcomes; PTSD symptoms decreased more in the intervention group; parents reported more externalizing difficulties, and teachers reported higher SDQ impact scores for the intervention group.
Mom et al. (2019)	Promotion‐oriented Capoeira Angola is an Afro‐Brazilian martial art that incorporates dance	36 weeks	Not mentioned	External instructor	Despite significant reductions in problematic behavior and increases in prosocial behavior, no significant reduction in teacher‐rated SDQ impact scores was observed, although a consistent trend was noted.
Peltonen et al. (2022)					
INSETT program	INSETT: Promotion‐oriented; aims to strengthen teachers' competence and self‐efficacy Manual based	Not specified	Not mentioned	Teacher	INSET: The intervention had no effect on internalizing, externalizing, prosocial behaviors, or resilience, but immigrant students' outcomes varied based on stressors and discrimination: high daily stressors led to increased externalizing problems, while discrimination increased prosocial behaviors in immigrants but decreased them in Finnish‐born youth.
PIER program	PIER: Promotion‐oriented; intervention program, social interaction, and group session Manual based	Not specified	Not mentioned	Teacher	No effect of the PIER program.
Quinlan et al. (2016)	Promotion‐oriented Creative arts therapy program, Home of Expressive Arts and Learning (HEAL)	10 weeks	Offered in the majority language	External professionals	No overall effect on the total SDQ difficulties score or most individual symptoms. However, the intervention did lead to a significant reduction in emotional symptoms in the intervention group compared to the control.
Rousseau et al. (2012)	Drama and language awareness‐based approach	12 weeks	Offered in the native language	External intervention team + teacher	No overall effect on symptom scores, but the experimental group did show a significant reduction in impairment. Some subgroups, like those exposed to promigratory violence or without school difficulties, showed slight improvements.
Rousseau et al. (2007)	Promotion‐oriented drama workshop	10 weeks	Offered in the native language	External drama project team	No overall effect on emotional and behavioral symptoms, self‐esteem, or language course grades. However, some improvements were observed in the intervention group, such as lower reported impact of difficulties (except for learning) and a significant improvement in math scores.
Rousseau et al. (2005)	Promotion‐oriented art therapy	12 weeks	Offered in the majority language	Teacher	Improvements were seen in emotional well‐being, perceived popularity, and a reduction in anxiety and depressive symptoms.
Spaas et al. (2023)					
CD program	Promotion‐oriented classroom drama (CD)art therapy‐based Manual based	9 weeks	Not mentioned	External drama therapists	CD: The intervention group saw increased perceived family support, while the control group reported a decline; friend support rose in the UK intervention group, but no overall effect was found; no significant impact on mental health, though higher baseline resilience helped buffer negative changes.
WTS program	Promotion‐oriented Welcome To School (WTS) Manual based	14 weeks	Not mentioned	Teacher	WTS: Mental health and social relations showed no significant effects, likely due to COVID‐19‐related school closures disrupting the intervention.
Tubbs Dolan et al. (2022)	Promotion‐oriented Social‐emotional learning	16 weeks	Offered in the native language	Teacher	HCT Program: Significant improvements were seen in teacher support, school climate, inclusivity, behavior, and subtraction skills, while literacy and numeracy remained unchanged.
Yankey and Biswas (2019)	Promotion‐oriented life skills training based on Bandura‚ Äôs (1986) social learning theory Manual based	Not mentioned	Not mentioned	Researchers, together with school staff	The intervention group reported significantly higher levels of active coping, emotional intelligence, and internalizing coping than the control group, but there was no effect on withdrawal coping, and the intervention group had lower self‐confidence.

All studies were published after 2000 and reported findings from an overall sample of *n* = 6316 participants. Although the majority of the study samples comprised recently arrived adolescents, some included participants with a wider age range, including children below age 12 (Fox et al. 2005; Rousseau et al. 2005; Spaas et al. 2023; Tubbs Dolan et al. 2022). Even though the main focus of the current scoping review was to identify studies on recently arrived immigrant adolescents residing in the resettlement country for up to 5 years, a few of the studies included participants who were living in the resettlement country for a longer duration, together with the newcomers (e.g., Spaas et al. 2023). Because the composition reflected ordinary school demographics, these studies were retained in the review. The studies from Australia, Europe, and North America (except one study) included participants who migrated from multiple countries with different ethnic/cultural backgrounds, whereas the studies from the majority world focused on migrant adolescents from the same country (Gormez et al. 2017; Tubbs Dolan et al. 2022; Yankey and Biswas 2019). On the other hand, a majority of the study participants were from Asia (e.g., Afghanistan, Pakistan, Tibet), South‐East Asia (e.g., Bhutan, Burma, Cambodia, Vietnam), the Middle East (e.g., Iraq, Syria), or the African continent (e.g., Eritrea, Ethiopia, Somalia, Sudan).

### 5.2 Programs Characteristics

The interventions reviewed were designed to address various psychosocial challenges faced by the refugee and immigrant population. The majority of the intervention programs (*n* = 11) were promotion‐oriented, focusing on promoting a sense of belonging and improving life skills development. Conversely, a total of five intervention programs were prevention‐oriented, focusing on addressing trauma‐related symptoms, stress, and behavioral problems (Baker and Jones 2006; Cardeli et al. 2020; Fox et al. 2005; Gormez et al. 2017; Kevers et al. 2022). These included CBT programs in Turkey (Gormez et al. 2017) and in the United States (Fox et al. 2005) and the Trauma Systems Therapy program in the U.S. (Cardeli et al. 2020). Most programs aimed to promote social‐emotional well‐being and mitigate mental health problems (e.g., Baker and Jones 2006; Tubbs Dolan et al. 2022), strengthen resilience and social support (Peltonen et al. 2022; Spaas et al. 2023), or address trauma‐related symptoms among highly vulnerable populations (*n* = 3) (e.g., Gormez et al. 2017; Mom et al. 2019). Others had a broader goal of fostering social cohesion and integration through creative and innovative approaches such as drama‐based interventions (*n* = 5) (e.g., Kevers et al. 2022; Rousseau et al. 2005).

Program duration ranged from as short as 5 weeks (Baker and Jones 2006) to 44 weeks, spanning over the whole academic year (Mom et al. 2019; Yankey and Biswas 2019). The variations in program duration were related to the intervention type. Programs using life‐skills training (Yankey and Biswas 2019), and socio‐emotional learning approaches (Tubbs Dolan et al. 2022) typically spanned over the whole academic term or year and had the longest implementation duration. Otherwise, the typical length of the programs was between 8 and 12 weeks, with a median duration of 8 weeks. Around half of the interventions were manualized programs (*n* = 9), whereas the programs based on art‐, drama‐, music‐therapy, or sports activities did not use a manual that could guide and structure the implementation process.

Out of the 17 interventions included in this review, five explicitly stated that they were delivered in the participants' native language to ensure accessibility and cultural appropriateness (e.g., Gormez et al. 2017; Rousseau et al. 2005, 2012; Tubbs Dolan et al. 2022; Fox et al. 2005). Another five interventions were implemented in the majority language of the host country (e.g., Cardeli et al. 2020; Quinlan et al. 2016). Notably, seven studies did not provide details on the language of delivery (e.g., Yankey and Biswas 2019; Mom et al. 2019), leaving it unclear how language barriers were addressed, if at all. The facilitation of interventions also varied significantly across studies. The programs were either implemented by only school teachers (*n* = 8), by researchers in collaboration with teachers (*n* = 2), or by external personnel, including researchers, therapists, or university students.

Although most of the studies (*n* = 9) did not mention any challenges with the implementation process of the programs (which may reflect gaps in reporting rather than the absence of such issues), some of the studies consistently reported challenges primarily related to engagement and participation. For example, low attendance and high attrition were highlighted in five studies (e.g., Tubbs Dolan et al. 2022; Spaas et al. 2023; Peltonen et al. 2022), where low participation rates could limit the effectiveness of the interventions (Tubbs Dolan et al. 2022), or complicate the analyses in general or bias the comparisons between intervention and control groups (Peltonen et al. 2022; Tubbs Dolan et al. 2022). Although attrition is often observed in intervention studies, special circumstances such as the COVID‐19 pandemic aggravated the level of attrition (e.g., Spaas et al. 2023). Finally, researchers faced difficulties in tracking participants for follow‐up assessments, resulting in an inability to assess longer‐term intervention effects. Common reasons for loss at follow‐up were changing classes or moving to different schools (e.g., Rousseau et al. 2005, 2007).

### 5.3 Evidence of Program Effectiveness

Both programs utilizing a CBT approach demonstrated the expected outcomes among participants (Fox et al. 2005; Gormez et al. 2017). The participants in these programs showed significant improvements in internalizing and emotional problems, such as depression and anxiety symptoms. Nevertheless, both studies evaluating a CBT approach used a single‐group design. Thus, these studies cannot reliably eliminate the possibility of alternative explanations for the observed change in symptom levels due to the lack of a control condition (Marsden and Torgerson 2012).

Two of the art therapy programs included in the review reported significant improvements in some of the measured outcomes. For example, Rousseau et al. (2005) reported improvements in perceived popularity and a reduction in anxiety and depressive symptoms among the participants of their art‐therapy program. Similarly, Spaas et al. (2023) reported an increase in perceived family support among the participants of their classroom drama program. However, they observed no improvements in other outcomes such as friend support, internalizing and externalizing symptoms, and PTSD symptoms (Spaas et al. 2023). Another art‐therapy‐based program (Kevers et al. 2022) reported no main effect, but conditional effects of the program. For example, participants who reported higher mutual peer relations at baseline further improved in mutual peer relations at post‐test. In addition, youth who displayed borderline or clinical levels of PTSD at baseline showed a decrease in symptom levels more than those in the control condition. On the other hand, no main effect of the program was observed for self‐reported, teacher‐reported, or parent‐reported mental health outcomes. In sum, the available studies generally yielded mixed findings regarding the effectiveness of art‐therapy‐based programs.

Among the life skills programs, half of the studies reported significant improvements in outcomes related to self‐worth, self‐concept, coping skills, and emotional regulation (e.g., Hannover et al. 2020; Peltonen et al. 2022, but only for the INSETT program). However, only one program (Tubbs Dolan et al. 2022) demonstrated significant improvements across a broad range of outcomes, including social and behavioral changes. The remaining programs did not report notable improvements in outcomes such as peer networks (Hannover et al. 2020) and sense of school belonging (Cardeli et al. 2020). Overall, these programs did not improve mental‐health outcomes. On the contrary, one of the studies even suggested an iatrogenic effect such that internalizing symptoms among the participants of the intervention group increased relative to the control group youth (Yankey and Biswas 2019).

Among the five programs based on drama, role‐play, music, or dance—which comprised one‐fourth of the studies— most did not demonstrate a statistically significant overall effect (e.g., Peltonen et al. 2022; Rousseau et al. 2007, 2012). While participants showed high engagement in these programs and some trends were observed, no measurable improvements were found in emotional, behavioral, or mental health outcomes.

Overall, the evaluation of the reviewed school‐based interventions offered some promising findings regarding the effectiveness of CBT‐based programs and a few of the art‐therapy programs in improving recently arrived adolescents' mental health outcomes. However, the reviewed studies provide limited evidence on the effectiveness of these programs in promoting social, behavioral, and academic adjustment and the well‐being of youth.

### 5.4 Methodological Features of Program Evaluations

Overall, cluster‐randomized and non‐randomized controlled designs were the primary choice of evaluation designs. Over one‐third of the studies (38%) included in the review used cluster‐randomized controlled designs. The remaining studies used either a non‐randomized design with a control condition (31%) or a single‐group pre‐ and post‐test design (25%). Around half of the studies (44%) were efficacy trials, in which researchers had strong control over the implementation process, whereas 38% were effectiveness studies, which mimicked testing the interventions in real‐life school conditions as closely as possible. The remaining 25% of the studies were pilot/feasibility tests of the interventions. The sample size of the studies varied between *n* = 31 and *n* = 4598. Gender distribution was relatively even at pre‐test across two‐thirds of the studies, although a few studies had either predominantly female (e.g., Baker and Jones 2006; Gormez et al. 2017) or male participants (e.g., Quinlan et al. 2016; Spaas et al. 2023). Only two of the studies had sample sizes as large as *n* = 995 (Peltonen et al. 2022) and *n* = 4598 (Tubbs Dolan et al. 2022). On the other hand, 7 studies had sample sizes between 31 and 58, and 8 studies had sample sizes between 108 and 318. The studies used various strategies to assess program outcomes. However, the predominant strategy (73%) was to assess participants before and after the intervention. Only three studies (20%) included a follow‐up assessment beyond the end of the intervention. One of the studies obtained up to five repeated assessments during the program implementation period, but did not include any follow‐up assessment (Baker and Jones 2006).

Although the use of randomized controlled trial design or inclusion of a control group in the evaluation of program outcomes were among the important strengths of the reviewed studies, several of the articles displayed deficiencies in the reporting practices. For example, the description of the program participants lacked some key information such as age range of the study participants (Baker and Jones 2006; Hannover et al. 2020; Peltonen et al. 2022; Quinlan et al. 2016), the average age of the sample (Rousseau et al. 2005, 2007; Yankey and Biswas 2019), or even the gender distribution (Yankey and Biswas 2019). Also, some did not mention how long the participants have been living in the host country (Fox et al. 2005; Peltonen et al. 2022; Rousseau et al. 2005; Tubbs Dolan et al. 2022; Yankey and Biswas 2019), or the language of the intervention (Kevers et al. 2022; Mom et al. 2019; Peltonen et al. 2022; Spaas et al. 2023; Yankey and Biswas 2019).

Several of the studies had weaknesses in the reporting practices of their findings. First, some of the studies did not present basic descriptive statistics for the key variables, which makes it difficult to evaluate how the participants scored on the main outcome measures (Cardeli et al. 2020; Yankey and Biswas 2019). In addition, there was a lack of clarity in the extent of support for some conclusions. For example, Cardeli and her colleagues (2020) argued that they observed a steep improvement among students with elevated levels of PTSD and depressive symptoms; this conclusion was not supplemented by any statistical analyses other than visual inspection of the descriptive statistics. Some other studies had ambiguities in their interpretation of data, which may potentially lead to inaccurate conclusions regarding the intervention effect. For example, Baker and Jones (2006) concluded that their music therapy program reduced externalizing behaviors and could be “used as an alternative to medication and other forms of school discipline” (p. 258). Nevertheless, the inspection of the descriptive table for the main program outcomes displayed no consistent change in the mean values of the key measures in the expected direction between the first and the last measurement points.

The reviewed studies showed substantial variations in the use of measurement tools that were appropriate for the linguistic and cultural background of the participants. Only one study explicitly clarified the use of measures validated for the intervention participants (Tubbs Dolan et al. 2022), whereas two studies did not provide any information about whether any linguistic or cultural adaptation was applied (Baker and Jones 2006). A typical approach was to use established measures that were previously used with different ethnic groups (e.g., Gormez et al. 2017; Mom et al. 2019; Rousseau et al. 2007). Nevertheless, these studies did not clarify whether the measurement tools were evaluated for their psychometric properties and validity within their target groups. A few studies translated their measures into the target group's language for linguistic adaptation; however, they did not reflect on whether the measures were relevant for the age and cultural background of the target group (e.g., Hannover et al. 2020; Rousseau et al. 2007, 2012). Hence, it is difficult to assess the quality of translated measurement tools without a thorough psychometric evaluation (Beaton et al. 2000). One notable approach to increase measurement quality was utilizing bilingual teachers to enhance participants' understanding by leveraging their linguistic abilities during data collection (Fox et al. 2005). In sum, the reviewed studies showed limited consistency in using valid and reliable measures appropriate to the linguistic and cultural backgrounds of the intervention participants.

## 6 Discussion

This scoping review aimed to compile and evaluate the school‐based programs designed to promote adjustment and well‐being among recently arrived immigrant adolescents in formal school contexts, where they spend a substantial amount of their time and can be easily reached. The findings reveal several important patterns regarding the current state of evidence, while also highlighting critical gaps in the literature that need to be addressed.

### 6.1 Evidence of Program Outcomes

The effectiveness of the programs varied substantially across different forms of interventions. The highest consistency in effectiveness was observed for the CBT‐based programs in leading to reductions in internalizing problems such as depression and anxiety symptoms (Fox et al. 2005; Gormez et al. 2017). This pattern is broadly consistent with meta‐analytic evidence showing that school‐based CBT yields moderate effects for anxiety and small‐to‐moderate effects for depression in youth (Mychailyszyn et al. 2012). Reviews focused on refugee and asylum‐seeking children also suggest promising, though mixed, effects of CBT and related psychosocial approaches, underscoring the need for more rigorous designs (e.g., Cowling and Anderson 2023). Although these two programs (Fox et al. 2005; Gormez et al. 2017) have shown consistent effects, both studies used a single‐group design, which has major shortcomings in attributing the observed changes to the intervention effect. The observed changes in single‐group designs could be due to factors unrelated to the program, such as the role of testing, maturation, and regression to the mean (Marsden and Torgerson 2012). Accordingly, future evaluations should prioritize controlled or randomized designs to strengthen causal inference.

The programs that used art therapy and life skills promotion approaches demonstrated mixed results across the studies. Some of the programs showed improvements in specific domains like self‐worth, coping skills, and perceived family support (Hannover et al. 2020; Peltonen et al. 2022). Other programs reported no significant effects or even potential iatrogenic effects such as increased school stress (Tubbs Dolan et al. 2022) or internalizing symptoms (Yankey and Biswas 2019). Although about half of the reviewed studies that used art therapy or life‐skills promotion approaches reported some promising outcomes, it should be noted that these studies also reported a significant number of null effects on the intended outcomes (Cardeli et al. 2020; Kevers et al. 2022; Peltonen et al. 2022; Spaas et al. 2023; Tubbs Dolan et al. 2022). From a developmental perspective, the content of life‐skills and SEL‐oriented programs align with core developmental tasks in adolescence (e.g., self‐regulation, peer skills). In addition, large‐scale meta‐analyses show that SEL programs can improve socioemotional skills, behavior, and academic performance when implemented with high quality and sufficient dosage (Durlak et al. 2011). In recently arrived youth, however, effectiveness may depend on cultural responsiveness and alignment with acculturative stressors experienced by the target group. In fact, the broader psychotherapy literature indicates that culturally tailored delivery and the competence of program leaders can facilitate engagement and outcomes among ethnically diverse youth (Huey et al. 2014). Thus, future research should go beyond “whether” these programs work and focus on examining “how” and “for whom” these programs work in line with the broader calls for testing program theory and components (Özdemir and Giannotta 2014).

A common approach in school‐based programs for recently arrived immigrants was using drama, role‐play, and music as key components of the intervention. These approaches were probably motivated by the successful use of drama‐based instructional methods in schools and their demonstrated effects on achievement outcomes (see Lee et al. 2015 for a meta‐analysis). Indeed, meta‐analytic work indicates drama‐based teaching is associated with positive effects on academic and psychosocial outcomes in general K–12 populations, especially when teacher‐led and delivered over multiple sessions (Lee et al. 2015). Nevertheless, our results suggest that the programs using drama, role‐play, and music as main components did not demonstrate statistically significant overall effects despite their theoretical promise and high participant engagement (Peltonen et al. 2022; Quinlan et al. 2016; Rousseau et al. 2007, 2012) unlike other reviews suggesting the effectiveness of such interventions in improving mental health in general populations (Jiang et al. 2023). An acculturative‐developmental explanation for the ineffectiveness of these programs may be plausible. Recently arrived immigrant youth may experience elevated levels of acculturative stress and greater difficulties with language, which may hinder their ability to engage in programs that demand active verbal involvement. Although drama and role‐play, especially when facilitated by trained teachers, can promote learning outcomes (Lee et al. 2015), limited language skills of the students, or the inability to deliver such programs at high quality without linguistically competent facilitators, could cause these approaches to fail in demonstrating the expected outcomes. Overall, these findings resonate with an ecological lens in which schools —as key microsystems— must adapt practices, language supports, and adult facilitation to optimize proximal processes for immigrant youth (Mehdipour Maralani and Pfeiffer 2025).

### 6.2 Methodological Qualities

Regarding methodological quality, the reviewed programs generally used highly praised methods in prevention science, such as randomized controlled and quasi‐experimental designs. These designs are potentially the most effective methods to elucidate the effects of interventions on intended outcomes (Shadish et al. 2002). Nevertheless, the reviewed studies suffered from several limitations that impact the quality of evidence. First, most studies were conducted on limited sample sizes, which reduces their statistical power (Hoyle and Gottfredson 2015). A small sample size is justifiable for evaluation studies focusing on special groups such as recently arrived immigrants and refugees. However, potential biases due to limited sample size should be addressed by using analytic strategies that may help researchers to develop a deeper understanding of the changes (Hopkin et al. 2015), or these biases could be mitigated by employing mixed‐methods research designs that combine both qualitative and quantitative methods to study program outcomes (Palinkas et al. 2019).

Another important limitation observed in the reviewed studies concerned their methodological qualities, specifically reporting practices. Several studies did not provide descriptive statistics and even some key demographic characteristics (e.g., Baker and Jones 2006; Cardeli et al. 2020; Quinlan et al. 2016; Yankey and Biswas 2019). There were even issues with disclosing information regarding the language of intervention or data collection (e.g., Mom et al. 2019; Yankey and Biswas 2019). In addition to a lack of specific information, there were also issues with reporting statistical estimates to support conclusions (Cardeli et al. 2020) or inaccurate interpretations of the findings (Baker and Jones 2006). The quality of reporting in these studies could be easily improved by following well‐received reporting guidelines such as CONSORT for randomized trials (Begg 1996) and STROBE for observational studies (Von Elm et al. 2007).

A critical methodological concern surfaced regarding the cultural and linguistic appropriateness of the measurement tools. All reviewed studies highlighted a lack of or limited language skills as one of the main challenges experienced by recently arrived immigrants, as well as a key factor that negatively contributes to their adjustment and well‐being. Despite these arguments, the linguistic backgrounds of the target groups were poorly addressed in most studies. For example, only one study explicitly reported using validated measures for their target population (Tubbs Dolan et al. 2022), while others either provided limited information about measurement adaptation or relied on previously used instruments without proper cultural validation. In addition, some of the studies ironically did not even mention the language in which the programs were delivered (Kevers et al. 2022; Peltonen et al. 2022; Spaas et al. 2023). In some instances, the program was delivered in the majority language, although the participants of the program had been living in the host country for a very limited period (e.g., Cardeli et al. 2020). Overall, null findings that were observed in some studies could be due to the use of linguistically inappropriate measurement tools or the delivery of the program in a language in which the participants are not sufficiently competent.

An overview of the intervention outcomes suggests a geographical concentration of research. The current literature offers a very limited understanding of evidence‐based interventions that could be used in school contexts in regions that host substantial immigrant and refugee populations. Most published intervention research on recently arrived immigrants and refugees originates from the WEIRD countries (i.e., Western, Educated, Industrialized, Rich, and Democratic) (Henrich et al. 2010), although these countries host around half of the international migrants and less than 30% of the forced migrants (UNHCR 2023). This disproportional distribution of research and migrant populations could be related to the availability of resources for and policies about supporting migrant populations in the country of resettlement. High‐income countries often adopt comprehensive policies and support mechanisms aimed at facilitating integration of newcomers (OECD 2023), which may provide more optimal conditions for offering different types of interventions in or outside of the school context. However, the current findings also showed that most interventions implemented in high‐income countries failed to demonstrate program impact, whereas all three programs implemented in low‐income countries showed significant improvements in the targeted outcomes. This pattern might partly reflect publication bias rather than the actual differences in program efficacy. In general, psychological and social science studies from low‐ and middle‐income countries face structural barriers to publication, including language constraints and limited access to high‐impact journals (Tindle 2021). Null or modest intervention effects could be less likely to be published, while significant results from underrepresented regions gain more visibility (Kühberger et al. 2014). On the other hand, researchers from high‐income countries may have greater opportunities to publish null findings in peer‐reviewed journals.

Another potential explanation for observing null findings in most studies from high‐income countries, but significant effects in studies from low‐income countries, could be related to the broader context of reception. Structural resources and supportive policies often promote the availability of psychosocial interventions. Generally, these contextual characteristics are expected to positively impact the effectiveness of interventions. However, in disadvantaged contexts, where adversities are prevalent and resources are limited, psychosocial interventions may be more effective than those offered in more advantaged settings (Kapetanovic et al. 2022) because these interventions may compensate for the lack of contextual support. Similarly, in countries with inclusive policies, children and youth often have access to services such as education, language support, and psychosocial care. In these contexts, school‐based interventions may complement existing provisions and demonstrate modest additive effects. In contrast, in countries where structural supports are limited or exclusionary, interventions may serve as critical protective factors against systemic barriers that undermine adjustment. In these settings, psychosocial programs may exert greater influence on children and youths' daily experiences and overall well‐being than in contexts where youth already have access to robust support systems (Barbui et al. 2020; Wong et al. 2025). In sum, macro‐level conditions may influence the availability and accessibility of school‐based services and shape observed intervention effects by determining the baseline against which program benefits are measured.

### 6.3 Implications for Future Research

This review identified several gaps and weaknesses that may guide future research aimed at promoting the positive adjustment and well‐being of recently arrived youth through school‐based programs. Overall, the reviewed studies showed limited attention to the mechanisms of change. A key priority for future research is to examine the mechanisms of change and the effectiveness of different components of the programs to better understand how and why programs do or do not lead to the expected outcomes (Özdemir and Giannotta 2014). These efforts may also provide explanations for why some theoretically promising pedagogical approaches (e.g., drama‐based programs) fail to show significant effects for recently arrived youth. Second, future research should focus on developing an understanding of how positive functioning in psychological, behavioral, and academic domains can be promoted using established or new approaches within a school context (Crooks et al. 2020; Mendes de Oliveira et al. 2022). An exclusive focus on mental‐health indicators, even in promotion‐oriented programs, hinders the development of knowledge on the promotion of well‐being and positive functioning. A potential strategy to overcome this narrow focus is to include program users (e.g., children, youth, families, and school personnel) in the program development process, as their involvement may enhance cultural sensitivity, improve alignment with the actual needs of the target group, and increase the program engagement and implementation quality, which may contribute to program effectiveness (Michael et al. 2023). A third priority is to monitor the participants beyond the completion of the intervention to understand the mid‐ and long‐term effects of these programs. Fourth, future studies should put more effort into following established reporting guidelines to increase transparency and provide a detailed description of the study design, measurement procedures, and implementation processes. Improvements in such areas can increase the quality of evidence. Next, future studies may benefit from adopting a clear focus on developing and testing programs that are feasible to implement using the existing infrastructure in schools and available personnel, such as school counselors and teachers, that are scalable, low‐cost, and do not require specialized clinical training. Such an agenda may increase the potential impact of research on practice and policy.

### 6.4 Limitations and Strengths

Several limitations and strengths of the current scoping review are worth mentioning. The current state of the literature has not provided much evidence on the effectiveness of programs that may promote the adjustment and well‐being of youth. Even the programs that explicitly aimed at promoting well‐being employed measurement tools that primarily focused on mental health and symptoms. Thus, there is limited evidence regarding the impact of these programs on the social, academic, and behavioral adjustment of recently arrived youth. Next, the current review could not provide an understanding of the program effects beyond the limited duration of the programs. The programs identified in this review almost exclusively assessed changes from pre‐ to post‐test following completion of the intervention, with a few exceptions (e.g., Fox et al. 2005; Peltonen et al. 2022). Therefore, it is unclear if the observed effects were retained over time or led to any longer‐term positive developmental outcomes.

The current review primarily included quantitative studies, at the expense of excluding qualitative studies, due to its focus on identifying the state‐of‐the‐art evidence on the effectiveness of programs for recently arrived youth. Qualitative methods have unrivaled strengths in providing insight into lived experiences, contextual factors, and implementation processes that quantitative studies may not capture adequately (Figueredo et al. 2013). Nevertheless, qualitative explorations cannot help researchers justify the program effect except in rare situations (Sechrest and Figueredo 1993; Shadish et al. 2002, 478). Including qualitative studies in this review could offer an understanding of how these programs are perceived and experienced by youth, educators, and families. However, such considerations were beyond of the scope of the current review.

The current review also focused exclusively on published evaluations in peer‐reviewed outlets, allowing mapping of evidence from studies that have passed the peer‐review process. This approach might have excluded some available evaluations in gray literature. However, there are mixed arguments about the role of gray literature in systematic reviews and meta‐analyses. For example, an analysis of 33 meta‐analytic studies showed that exclusion of gray literature in meta‐analyses may lead to overestimation of treatment and interventions effects (McAuley et al. 2000). On the other hand, another study found that inclusion of gray literature may also increase the bias in conclusions due to their overrepresentation of negative or null effects as well as the poor quality of research design and reporting practices (Martin et al. 2005). In sum, it is unclear how the inclusion of gray literature would alter the conclusions of the current scoping review.

Despite these limitations, several strengths of the current review should be highlighted. First, this study followed established scoping review methodologies (Arksey and O'Malley 2005; Munn et al. 2022) and adheres to PRISMA‐P guidelines (Moher et al. 2015). The scoping review methodology allowed a broader mapping of existing literature on school‐based programs that focused on various types of outcomes and relied on different study designs to evaluate their impact. This field of research has not produced enough studies with a similar focus and characteristics, which could be compiled and evaluated in‐depth using a systematic review or meta‐analytic approach. Another important strength of the study is the exclusive focus on school‐based interventions delivered in a formal‐schooling setting. Unlike other existing reviews, we focused on compiling evidence on the potential of school‐based programs in promoting positive functioning among recently arrived adolescents. The review also screened the null and iatrogenic effects of the studies to provide a more accurate understanding of the program outcomes by presenting both expected and unexpected findings together.

## 7 Conclusion

This study presented a scoping review of the evidence regarding the effectiveness of school‐based programs in promoting positive functioning and well‐being among recently arrived immigrant adolescents. Although this review identified several programs focused on promoting the adjustment and development of recently arrived adolescents, it was revealed that these programs were far from providing consistent evidence of their effectiveness. More importantly, most of these studies adopted a mental health focus rather than acknowledging and prioritizing the developmental challenges and acculturative needs of the resettling youth (Jugert and Titzmann 2020; Motti‐Stefanidi et al. 2012). In addition, the available studies suffered from several methodological limitations. These include the use of small samples, very limited follow‐up beyond post‐test assessment, inconsistent consideration of the linguistic competencies of the target group in the measurement of program outcomes and in program implementation, low transparency, and poor reporting practices. Overall, the findings were mixed and provided limited high‐quality evidence to support the dissemination of any particular intervention mode to promote the adjustment and well‐being of youth in school settings. Therefore, researchers are encouraged to develop and test interventions that focus on the developmental and acculturative needs of youth, especially in regions where a substantial number of international immigrants resettle. They should also align program implementation and research procedures with the cultural and linguistic background of the target group as well as the school context, use established reporting practices, and, more importantly, focus on why, how, and for whom these interventions lead (or not) to expected outcomes. These concentrated efforts may pave the way for the development of effective and scalable interventions. Such interventions can be implemented with ease using existing school resources and, in turn, contribute to the overall positive functioning of recently arrived immigrant youth.

## Author Contributions

M.Ö. and B.O. developed the research questions, the review protocol, inclusion and exclusion criteria with major contributions from S.B.Ö. M.Ö., together with S.A.N., L.A., and H.A. managed the study selection and data extraction, and writing the manuscript. B.O. and S.B.Ö. critically reviewed the manuscript and contributed to the editing and rewriting processes.

## Ethics Statement

Ethical review of the research protocol is not necessary because the current scoping review does not process or report on any personal data.

## Supporting information

Appendix.

## Data Availability

The data that support the findings of this study are available from the corresponding author upon reasonable request.
