# Supplementary material for: A Scoping Review of School‐Based Programs for Promoting Recently Arrived Immigrant Youth's Positive Adjustment and Well‐Being
Source: J Community Psychol. 2026 Feb 10;54(2):e70088. doi: 10.1002/jcop.70088 (PMC12890287; doi:10.1002/jcop.70088)
Supplement: Supplementary file 1 — Appendix. [file JCOP-54-0-s001.docx]

**APPENDIX**

**Database Search Method**

The following strategy was applied to search PsychINFO. The keywords and steps were adjusted to search the other databased to meet the database-specific requirements. Search methods applied to other databases are available upon request from the corresponding author.

| **Steps** | **Search keywords** |
| --- | --- |
| 1 | exp Refugees/ or Asylum Seeking/ |
| 2 | ((newly or recently or unaccompanied) adj6 (refuge* or "asylum seek*" or immigrant or migrant? or "displaced person?")).tw. |
| 3 | ((displaced or refugee) adj3 (adolescen* or child* or juvenile* or minor or person*or people* or teen* or young or youth*)).tw. |
| 4 | 1 or 2 or 3 |
| 5 | Students/ or exp Adolescent Behavior/ or exp Adolescent Development/ or Student Characteristics/ |
| 6 | ((refugee or asylum seek* or unaccompanied or boy? or girl? or student?) adj8 (age* adj ("12" or "13" or "14" or "15" or "16" or "17" or "18" or "19" or "20" or "21" or "22" or "23"))).tw. |
| 7 | 5 or 6 |
| 8 | schools/ or education/ or multicultural education/ or special education/ |
| 9 | ("school-based" adj6 (program? or prevention or adjustment or achievement or grades or adaption or satisfaction or intervention? or treatment)).tw. |
| 10 | ((classroom or "class room" or education* or learn* or school*) adj3 (based or environment* or multicultur* or program* or implementation)).tw. |
| 11 | 8 or 9 or 10 |
| 12 | 4 and 7 and 11 |
| 13 | limit 12 to yr = "2000 -Current" |
| 14 | remove duplicates from 13 |
